# Milk Emulsions: Structure and Stability

**DOI:** 10.3390/foods8100483

**Published:** 2019-10-11

**Authors:** Katja Braun, Andreas Hanewald, Thomas A. Vilgis

**Affiliations:** 1 Department of Food Technology, University of Applied Science Fulda, Leipziger Str. 123, 36037 Fulda, Germany; braun.katja@gmx.net; 2 Max-Planck-Institute for Polymer Research, Ackermannweg 10, 55128 Mainz, Germany

**Keywords:** milk, emulsions, whey protein, casein, rheology, microscopy, rheo-optics, jamming transition

## Abstract

The main aim of this research is to investigate the characteristics of milk and milk proteins as natural emulsifiers. It is still largely unclear how the two main fractions of the milk proteins behave as emulsifier in highly concentrated emulsions. The surface-active effect of these is determined experimentally for emulsions with a high oil content (φ > 0.7), in this case fully refined rapeseed oil. Recent publications have not yet sufficiently investigated how proteins from native milk behave in emulsions in which a jamming transition is observed. In addition, scientific measurements comparing fresh milk emulsions and emulsions of dried milk protein powders based on rheological and thermal properties are pending and unexamined. The emulsions, prepared with a rotor-stator disperser, are investigated by their particle size and analysed by microscopy, characterised by their rheological properties. The behaviour under shear is directly observed by rheo-optical methods, which enables the direct observation of the dynamic behaviour of the oil droplets undergoing a size selective jamming transition. For a better understanding of the contributions of the different emulsifying proteins, oil-in-water emulsions have been prepared by using whey protein isolates and sodium casinates. Their different role (and function) on the interface activity can be assigned to the droplet sizes and mechanical behaviour during increasing shear deformation. In addition, solid (gelled) emulsions are prepared by heating. It is shown that the cysteine-containing whey proteins are mainly responsible for the sol–gel transition in the continuous water phase and the formation of soft solids.

## 1. Introduction

In the food and cosmetics industry, milk proteins are used as natural products in a variety of products [1]. The proteins contained in fresh milk are obtained by the membrane process or by precipitation [2]. As fresh milk and its proteins are natural products, no declaration with E-numbers is required, which, in turn, corresponds to the trend towards “clean labelling”. Milk proteins consist of two main fractions: caseins by about 80% and whey proteins by around 20%. The different structural and functional properties of the two fractions allow them to be used in a wide range of applications. Caseins are very heat stable, but denature at a pH value of approximately 4.6. Heat-sensitive milk proteins include whey proteins, which begin to denature partially at temperatures above 65 °C [3].

It is well known that milk-based proteins are excellent emulsifiers because of their amphiphilic structure [4]. The hydrophobic and hydrophilic parts of the proteins are able to connect a water and an oil phase and allow more detailed physical models, as, for example, reviewed in [5]. A stabilisation of the fat phase, due to oil droplet formation and stabilisation by milk proteins, can be achieved by different (mechanical) methods of emulsification. Classic rotor-stator systems, high-pressure homogenisers, ultrasonic methods or membrane processes can be used to produce stable emulsions. Even highly creamy mayonnaise-like emulsions can form when the added oil fraction is sufficiently high. The size of the oil droplets and ultimately the stability of the emulsion depend on these and on the shear rates [6]. Milk proteins are not only suitable as emulsifiers, but also as foam stabilisers. They can also be used as gelling agents, and they allow for solid and semi-solid milk emulsions [7], which will be addressed below in some detail. Milk is thus a perfect basis for stable natural emulsions with high oil content.

Little is known about the detailed physics and molecular mechanisms of milk emulsions. However, in this investigation, the main focus is drawn on the mesoscales around the droplet size, as most of the structural and dynamical information of the emulsions is defined on such length scales. Emulsions composed by milk and high vegetable oil concentrations (>65%) result in a mayonnaise-like texture. The viscosity of these emulsions increases due to lack of motion of the oil droplets in the continuous water (whey) phase. The strong viscosity increase is described by the “jamming transition” in which the oil droplets are very densely packed and trapped in a cage. A shape change from spherical oil droplets at lower concentrations to non-spherical oil droplets at larger concentrations can be observed [8]. The droplets become then caught in a cage formed by the surrounding particles. When this so called jamming transition is reached [9], the emulsion appears “creamy”, stable and “jelly” at low shear rates.

During emulsification milk proteins adsorb at the interface or at the surface of the oil droplets and form a thin layer. Completely denatured caseins and their fractions are considered more flexible and faster than whey proteins [10]. It is well known that whey proteins form cross-links under heating. When heat is gently applied, the emulsions form soft solids [11] with a very special mouthfeel.

Concentrated milk protein solutions, such as whey protein concentrate (WPC), whey protein isolate (WPI) and caseinate, are either acid or rennet precipitated caseins, make dosing easily possible. Sodium caseinate is widely used in industrial processes due to its high and quick water solubility. Sodium caseinates, whey protein isolates or whey protein concentrates are mainly used for emulsification because of their excellent surface-active properties [12,13].

In the following, the role and contributions to emulsion formation of fresh milk and different milk protein isolates will be investigated. Special focus is drawn on the emulsifying properties of fresh milk and reconstructed emulsions from whey protein isolated and caseinates. Moreover, here only, emulsions of the mayonnaise type will be investigated. In the case of fresh milk, this means oil contents higher than 68% in weight proportions. The aim of the mast study is to determine the physical molecular background of this effect experimentally by testing different oil contents. In particular, the thermal behaviour of emulsions which form soft solids is still largely unexplained.

In view of the increasing demand on “natural products”, the main focus of this paper is the investigation of the emulsion capability of native milk, that means pasteurised but nonhomogenised. Therefore, the main body of the present paper treats emulsions stabilised by native milk. In order to maintain the fat and micellar casein structure the emulsification process needs to be carried out with care. Therefore, the shear forces during emulsification need to be sufficiently small, but large enough to get stable emulsions. 

Different emulsions on the base of fresh milk, sodium caseinate (Na-Cas) and WPI with vegetable oils are prepared and compared to each other. Also, mixtures of Na-Cas and WPI will be investigated. In addition to the preparation of the emulsions with a rotor-stator system, this work includes the determination of the oil droplet size with a Laser Diffraction Particle Size Analyser. The comparison of the results obtrained from the Particle Size Analyser and the sizes obtained from light microscope provide the first insight on the emulsion structure. In parallel, the rheological properties of the milk emulsions are investigated and correlated with optical microscopy. Additionally, rheo-optics, i.e., the simultanous measurements of the moduli and the observation of the droplet motion, provides a consistent interpretation of the behaviour of the dense emulsions. Finally, the rheological measurement of the differently concentrated emulsions and the influence on the temperature play a central role for understanding the gel formation in the emulsions. 

As mentioned before, all emulsions under consideration have oil concentrations of 72 to 74% (*w*/*w*), and are sufficiently stable in the time range between 5 and 11 days. A visual observation of these emulsions, i.e., the detection of an oil film on the surface, is recorded at regular intervals by photographs. These observation are supported by a by a consequent analysis of the droplet size measured by the particle analyser. The comparison of all emulsions produced should elaborate on the properties of the individual milk protein fractions and their thermal properties in highly concentrated emulsions. The theoretical bases of the milk proteins, emulsions, methods and rheology provide methods to help understand the results and interpretations. The results and interpretation of the particle size analysis, light microscopy, rheology and rheo-optics/microscopy will provide a consistent physical picture of milk emulsions.

## 2. Materials and Methods

### 2.1. Materials

The materials for emulsion preparation and sample preparation are freshly prepared and consumed within one day. The fresh milk is used only in the period until the best-before date. For the preparation of milk emulsions, hay milk was used by the company “Gläserne Molkerei”. This milk was pasteurised by the manufacturer at 72–74 °C for 40 s and is not homogenised. In addition, this is a milk of organic quality. The rapeseed oil from Rapso was purchased from a local supermarket. This is a fully refined rapeseed oil, which has no, or only traces of, phospholipids and lecithins. It is commercially available in 0.75 L bottles. According to the test report from the producer, the product has an absolute density of 0.9173 g/cm³ and a smoke point of 217 °C. In the producer report, it was also mentioned that the oil was free of phospholipids (“lecithin”), which was the main reason the choice of the oil to prevent influences from different origin of emulsifiers.

The sodium caseinate from Sigma Aldrich (CAS number 9005-46-3) contains the casein fractions α_s1_-casein, α_s2_-casein, β-casein and κ-casein. In addition, it is water-soluble due to its salt and has a casein content of 80%. According to the data sheet it contained additionally nitrogen 13.5–16.0%, presumably in form of free amino acids, sodium (Na) ≤3% and the remainder water.

The whey protein isolate from Davisco, BiPRO, consists of the whey protein fractions β-Lactoglobulin and α-Lactalbumin and is not denatured as described. This product has a protein content of at least 95%.

The surfactant sodium dodecyl sulphate (SDS) is used to dilute all emulsions to measure in the Laser Diffraction Particle Size Analyser. The SDS was used by the company Roth with the CAS number 151-21-3. The low-concentrated SDS solution prevents cluster formation of the oil droplets in the water phase.

For the dilution and preparation of the protein solutions, ultrapure water Milli-Q^®^ water is used. The preparation of the emulsions and the performance of amplitude sweep and frequency sweep, with subsequent particle size measurement, were carried out within one day. The measurement of the temperature profile from 4 °C to 80 °C, and subsequent cooling from 80 °C to 4 °C is carried out within 3 days after cooling the samples in the refrigerator at 4 °C. A sample is taken from the centre of the emulsion.

### 2.2. Emulsion Preparation: Milk Emulsions

All emulsions were prepared with an ULTRA-TURRAX^®^ T 18 basic (rotor-stator principle) from IKA-Werke GmbH & Co. KG, Staufen im Breisgau, Germany. According to the manufacturer, with this mechanical principle it is possible to achieve a final fineness of the emulsion of 1–10 microns. All 50 g emulsions were run for 5 min at a speed of 15,600 rpm. The main intention to use this configuration was not to change the native stat of the milk, i.e., the membranes around native fat particles and the casein micelles of the nonhomogenised milk. 

### 2.3. Emulsion of Milk Proteins and Vegetable Oils

Emulsions of the respective sodium caseinate, whey protein isoate (WPI) or mixed sodium caseinate/whey protein isolate solution were made with MilliQ water. Thereafter, the desired oil concentration was emulsified with the protein solutions. The percentages are all in weight percent % (*w*/*w*).

### 2.4. Optical Microscope Observation

The light microscope Axio Scope.A1 from ZEISS was used to record micrographs. The images were taken using the principle of bright field microscopy. There was a 10-fold and 40-fold magnification used. For evaluation the software image was used to insert the scale bar.

### 2.5. Particle Size Distribution

To determine the oil droplet size, a Laser Diffraction Particle Analyser LS 13,320 from Beckman-Coulter, Düsseldorf, Germany was used, which also allows measurement of particles in the measuring range of 0.040 to 2000 μm. The underlying theory is the Fraunhofer model, which does not require any additional data, such as refractive index and absorption coefficients for the calculation. The model can be used without problems, as the shape of the oil droplets are perfectly spherical in this experiment.

For the measurement of the emulsions, a mixture of 1:9 (1 g sample + 9 g 0.2% SDS solution) is prepared beforehand. To avoid aggregation of the oil droplets in the water phase, dilution was carried out with a negatively charged surfactant. To prevent this effect or obtain perfect results of the oil droplet size, sodium dodecyl sulphate (SDS) was used. The sample was set immediately before the measurement and mixed at 1000 rpm for ~10 s. Each sample was measured three times.

Before each measurement, a calibration is performed to obtain the most accurate results possible. This includes the degassing or removal of air bubbles, measurement of the background, compensation and adjustment of the sample space. For all graphical and statistical evaluations, the software OriginPro 8.5 has been employed.

### 2.6. Measurement of Rheological Properties

For rheological measurement, the air-bearing rheometer Gemini 200 was used by Malvern Instruments (former Bohlin Instruments). The plate–plate geometry used by Malvern Instruments consists of a plate with a diameter of 25 mm. The plate gap for all samples analysed is 1 mm. A fourfold determination was made for the amplitude sweep and frequency sweep measurements. The entire graphical evaluation of the rheological tests has been performed in the graphics software OriginPro 8.5 also.

The amplitude sweep was used for studying the nonlinear mechanical response of the samples, which is relevant for structural changes. For all samples, the deformation γ was chosen from 0.1% to 100% with an angular frequency ω of 10 rad/s.

The frequency sweep starts at an angular frequency ω of 0.1 rad/s and ended at 100 rad/s while the constant deformation γ was 0.5% (linear regime). The value of the deformation was chosen from preliminary experiments in the LVE-range.

Temperature-dependent behaviour: To investigate the behaviour of the samples on the temperature, temperature ramps were set at 1 °C/min. The temperature profile was chosen from 4 °C to 80 °C and then cooled from 80 °C to 4 °C. The temperature profile is displayed in the results as the second y-axis. To prevent the sample from curing, insulation is required throughout the measurement. 

### 2.7. Statistical Analyses

For the evaluation, a check is made to determine whether there is a correlation between the variables protein concentration (at constant oil content) and the droplet size. From each of the triple measurements, the mean values (triple determination) are used for the correlation analysis. 

The calculation of the correlation coefficient, according to Bravais-Pearson, for metric data is given by

(1) $$r=\frac{\Sigma \;\left(x_{i}-\bar{x}\right)\left(y_{i}-\bar{y}\right)}{\sqrt{\Sigma \;}{\left(x_{i}-\bar{x}\right)}^{2}\;\Sigma {\left(y-\bar{y}\right)}^{2}}$$

## 3. Results

### 3.1. Stability of Emulsions

#### 3.1.1. Emulsion with Fresh Milk

**Figure 1:**
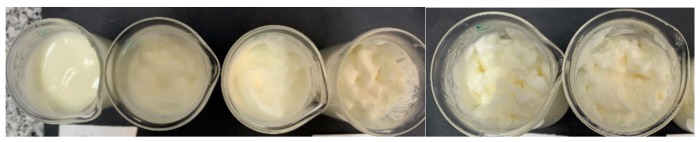
Stable emulsion of fresh milk containing 65%, 70%, 71%, 72%, 73% and 74% of oil (from left to right) after 6 days storage at 4 °C.

Emulsions of rapeseed oil and fresh milk from 65% to 74% *w*/*w* oil were investigated. All emulsions were produced at a room temperature of ~25 °C ± 1 °C and are shown in Figure 1.

Very unstable emulsions show oil films or visible free oil on the surface. The emulsions produced at different concentrations are shown in Figure 1. At oil concentrations from 70% *w*/*w* rapeseed oil and 30% *w*/*w* fresh milk, a pronounced creaminess has been achieved. The viscosity further increases by increasing oil concentration. With even higher oil concentration (>74%), the emulsions start to break down, even during the emulsion preparation. Temperature, as a second parameter, could also be a factor in this observation. The natural amount of emulsifiers in the fresh milk, consisting of free polar lipids, whey proteins, casein, plays an important role. With increasing oil concentration, the amount of emulsifiers for stabilising the oil droplets is insufficient. The latter is the main cause of unstable emulsions.

#### 3.1.2. Emulsions with Whey Proteins and Caseinates

**Figure 2:**
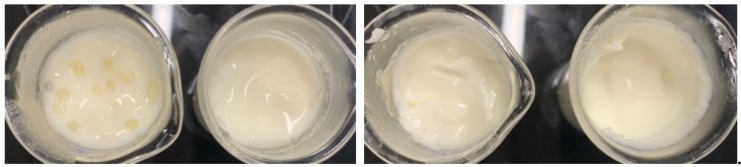
Left: Emulsion after 4 days: left: emulsion with 7% *w*/*w* whey protein isolate (WPI) solution; right: emulsion with 8% *w*/*w* WPI solution; oil content 73% *w*/*w*. Right: Emulsion after 4 days: left: emulsion with 7% *w*/*w* sodium caseinate solution; right: emulsion with 8% *w*/*w* sodium caseinate solution; oil content 73% *w*/*w*.

When sodium caseinate is used, an increasing creaminess was observed with increasing protein concentration in the aqueous phase. The experiment showed that the use of sodium caseinate is very well suited as emulsifier. Compared with the whey protein isolate emulsions, sodium caseinate emulsions showed better stability. The direct comparison is shown in Figure 2 for convenience.

The literature also confirms this observation of improved stabiliy with increasing sodium caseinate concentration [14]. The higher flexibility of the denatured caseins, caused by the isolation process, allows a faster development of its interface activity. Moreover, the higher hydrophobicity of the casein supports the higher stability of the emulsions, compared to those prepared by more hydrophilic whey proteins. Also, note that sodium caseinate or WPI emulsions are less sensitive to the mechanical impact of the shear by the Ultra Turrax, compared with the originally native proteins in fresh milk. 

The maximum emulsifying capacity in the case of emulsions with fresh milk is according to the here presented results between 71% *w*/*w* and 73% *w*/*w* rapeseed oil. When comparing literature values independent of the emulsion composition, Malkin reported a maximum packing of highly concentrated emulsions of 71.2% [15]. Depending on the droplet size, Derkack [16] also specifies a limited pack between 71% and 75%.

At an oil content of 75% *w*/*w*, a complete emulsification is no longer guaranteed. The emulsion broke due to lack of sufficiently available emulsifiers (whey proteins and caseins) in the native milk. To summarise these observations, fresh milk emulsions show good stability compared to emulsions with protein solutions at oil concentrations between 68% and 73% rapeseed oil. It has already been confirmed, after the preparation of emulsions, that milk proteins are also very suitable to produce highly concentrated, mayonnaise-like emulsions. Especially when fresh milk is used, emulsification by a stator-rotor system quickly results in emulsions with a solid-like behaviour.

### 3.2. Size of the Oil Droplets

**Figure 3:**
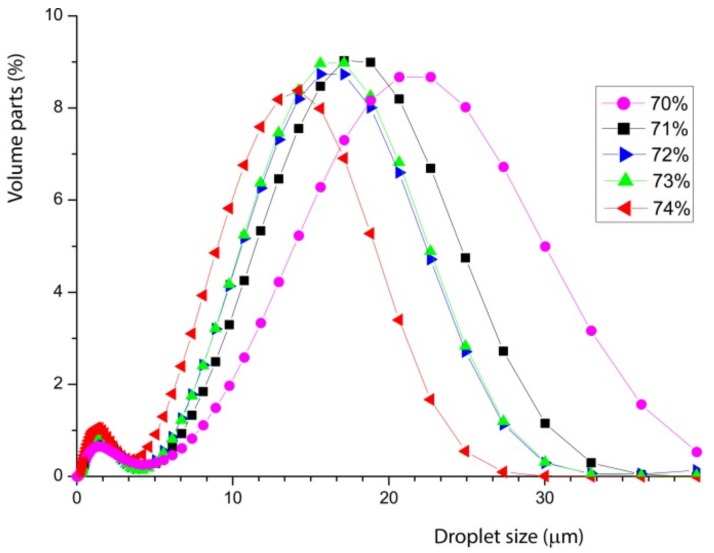
Oil drop size distribution of milk emulsions with varying oil proportions 70% *w*/*w* to 74% *w*/*w* and fresh milk.

One of the most important control parameter for the stability is the oil droplet size. When examining the oil droplet size in the Particle Size Analyzer, as shown in Figure 3, a slightly decreasing diameter is observed with increasing oil content in emulsions containing fresh milk. In addition, a bimodal size distribution with two maxima can be observed in all five emulsions. A bimodal distribution of the diameters can be seen in the fresh milk emulsions, as the native milk fat is still present in the emulsions. The droplets of the native milk fat are represented by the first small peak in Figure 3 and are significantly smaller in diameter than the emulsified rapeseed oil; of course, their size does not depend on the amount of added oil.

**Figure 4:**
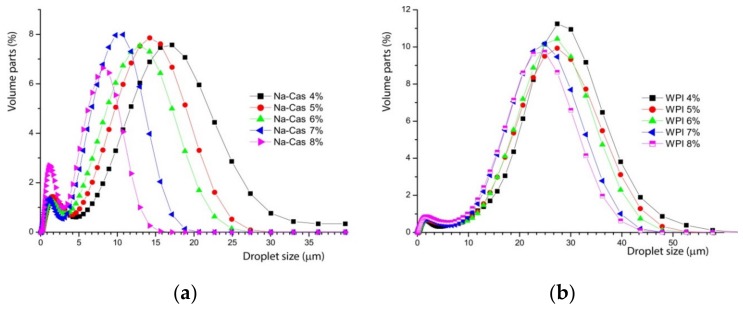
Oil drop size distribution of reconstructed emulsions from different concentrations of sodium caseinate, Na-Cas (**a**) and whey protein isolate, WPI, (**b**) at given oil concentration of 73% *w*/*w* with respect to water.

Note how the mean droplet size shifts systematically with the amount of added oil in the stable concentration regime. This becomes more systematic, when emulsions with whey protein isolate and casein are prepared. Figure 4 shows both emulsions with different sodium caseinate (Na-Cas) and whey protein isolate concentrations (WPI). All emulsions contain 73% *w*/*w* oil content, whereas the sodium Na-Cas and the WPI concentration vary systematically. This shows, in both cases, that by increasing the corresponding protein concentration, the oil droplet size decreases. 

The droplet size decreases with increasing concentration of emulsifiers. This is relatively simple to understand. As more emulsifiers are available, more surface area at a given volume can be occupied, by stabilising a larger number of smaller droplets. Obviously, the different proteins yield a very different droplet size. With a protein content of 8% *w*/*w* in the aqueous phase, the greatest shift and difference in the mean values are observed, as shown graphically in Figure 4. The mean values of the emulsions are shifted by a factor of three. The decreasing oil droplet size with increasing sodium caseinate concentration in the aqueous phase also applies to the range of highly concentrated emulsions by 73% *w*/*w* oil content. The literature confirms the quicker and enhanced emulsifier properties of caseins compared to whey proteins, too [4,12]. This is because of higher surface-active and faster adsorption of the caseins at the interface, which enable the rapid stabilisation of the resulting small oil droplets during the preparation of the emulsion. 

For visualisation, directly compare the different curves of Figure 4 at their highest concentration to recognise the significant differences.

**Figure 5:**
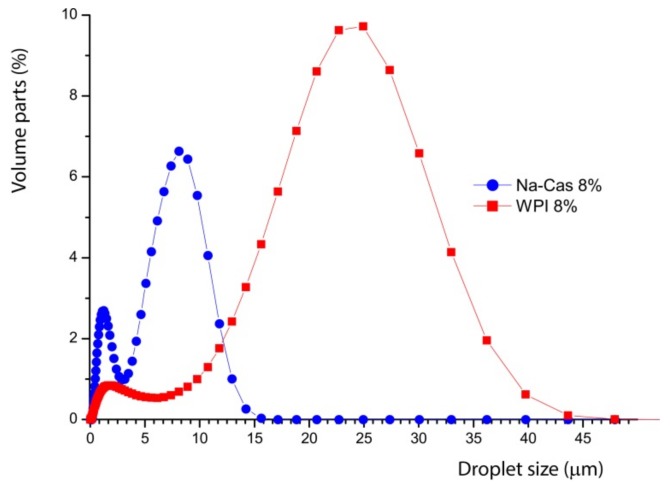
Oil drop size distribution of emulsion consisting of water and 73% oil emulsified with Na-Cas (blue) and WPI (red).

**Figure 6:**
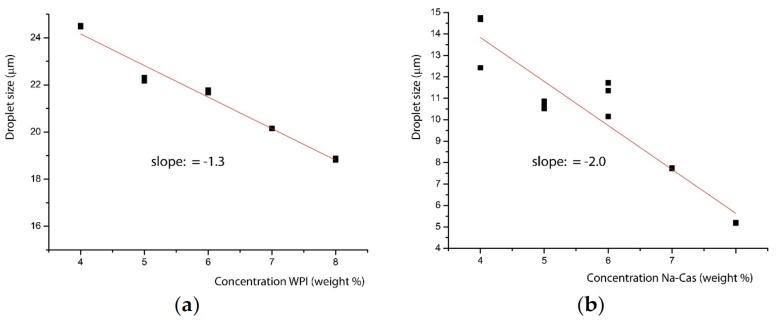
Linear fit of the decrease of the droplet size with increasing protein concentration. Left, (**a**) WPI; right, (**b**) Na-Cas. The slope in the case of Na-Cas is much steeper.

In Figure 5, it can be seen that the whey protein isolate yield differs significantly, by a factor of 3 larger oil droplets. This suggests very different molecular emulsification processes for the different proteins and will be discussed below. This observation has to be seen in the context of the scaling of the decrease of the particle size with increasing concentration of WPI and Na-Cas, which is shown in Figure 6.

In both cases, a strong negative correlation (r_WPI_ = −0.9859, r_Na-Cas_ = −0.9473) is shown, which again indicates that an increasing protein concentration results in decreasing droplet sizes. The strong negative correlation of protein concentration and oil droplet size is due to the adsorption mechanism, the arrangement of the different emulsifiers and the associated stabilisation mechanisms. The more milk proteins are present in the aqueous solution, and subsequent emulsification, the better the stabilisation of the oil droplets; these adsorb on the surface of the droplets and stabilise them sterically and electrostatically [17].

### 3.3. Microscopy

**Figure 7:**
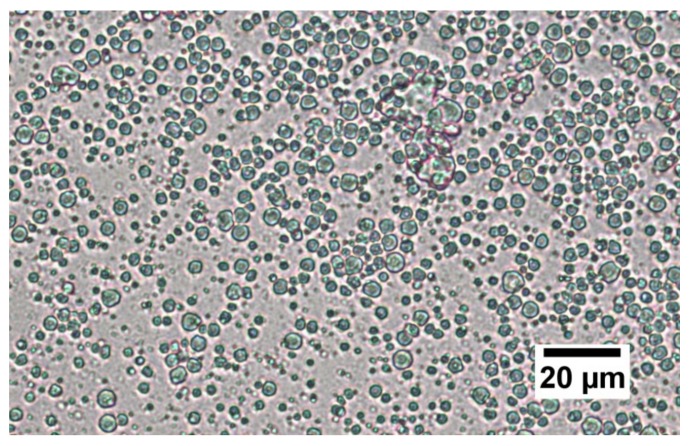
A typical microscopy picture of the natural organic hay milk used for the milk emulsions in this paper. The magnification is 40×.

Light microscopy will directly support the data from the particle size analysis and the structure of the different emulsions. The reference emulsions with fresh milk have been prepared with pasteurised and nonhomogenised milk, with its natural fat content. Due to pasteurisation, the casein micelles are still native, and, due to the nonhomogenisation, the fat particles are native as well. The fat droplets of natural milk are clearly visible under light microscopy, shown in Figure 7. 

The typical size of the native fat droplets in the nonhomogenised milk agrees somehow with the measurements in the particle analyser. These have also been measured separately (not shown in this paper). However, the same size appears as small peak for the reconstructed samples with WPI and Na-Cas. Therefore, to clarify the meaning of the peak at small diameters in Figure 4 and Figure 5, it useful to study microscopy.

**Figure 8:**
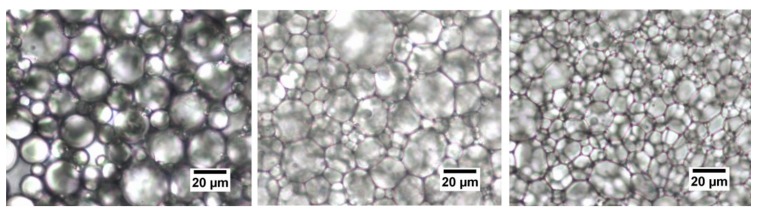
Emulsion with fresh milk with 65%, 70% and 73% oil (from left to right). The magnification is 40×.

A selection of typical microscopic pictures of the emulsions is shown in Figure 8.

In the microscopic images, the oil droplets are generally difficult to bring into focus due to their dense packing. Figure 8 shows three typical emulsions prepared with fresh milk with 65% *w*/*w*, 70% *w*/*w* and 73% *w*/*w* from left to right. The pictures confirm the broad size distribution of the oil droplets at any concentration, which goes back to the preparation method with the rotor-stator method. The surface active proteins adsorb at distribute themselves statistically at the interfaces, which are formed under nonequilibrium conditions, stabilise the oil droplets. Nevertheless, the microscopic pictures conform the findings from the particle analysis that the mean size of the droplet decrease by increasing oil concentration. It can also be seen that the shape of the droplets changes from spherical (65%) to polyhedral (70% and 73%). This effect turns out to be more pronounced at higher concentrations. Each droplet becomes more and more confined by its nearest neighbours, which indicates the jamming transition visually. A number of small, roughly concentration independent droplets (<10 µm) can be seen as well, which correspond to the peak in the particle size distributions shown in Figure 3.

**Figure 9:**
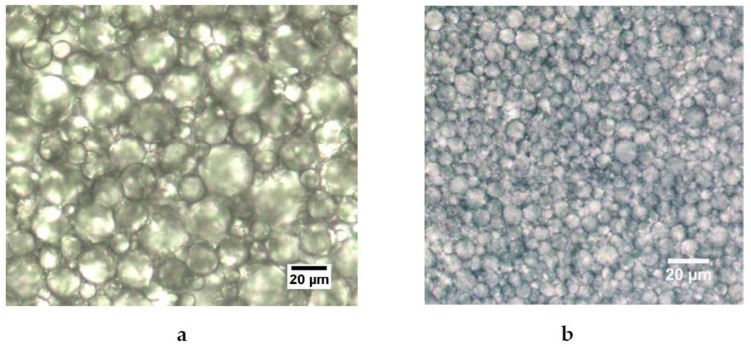
Optical micrographs of reconstructed emulsions of water and 73% *w*/*w* oil with WPI (**a**) and Na-Cas (**b**). The magnification is 40x.

The optical micrographs of the reconstructed emulsion also confirm the results measured in the particle analysis from Figure 5, see Figure 9a,b.

The emulsion prepared with WPI shows larger particles, whereas when Na-Cas is used as emulsifier, the oil droplets are much smaller, as already indicated in Figure 5. In both cases, a fraction of much smaller droplets is visible, as also suggested in Figure 5.

### 3.4. Rheology

The rheology of the emulsions provides further key information of the different milk emulsions; especially, the nonlinear behaviour will provide a deeper insight in the behaviour of the milk emulsions. For the purpose of this paper, amplitude and frequency sweeps have been performed. In addition, temperature ramps have been performed to provide more insight into the gelation behaviour of the different milk proteins. The chosen parameters for the rheological measurements are listed in Table 1.

#### 3.4.1. Amplitude Sweeps

**Figure 10:**
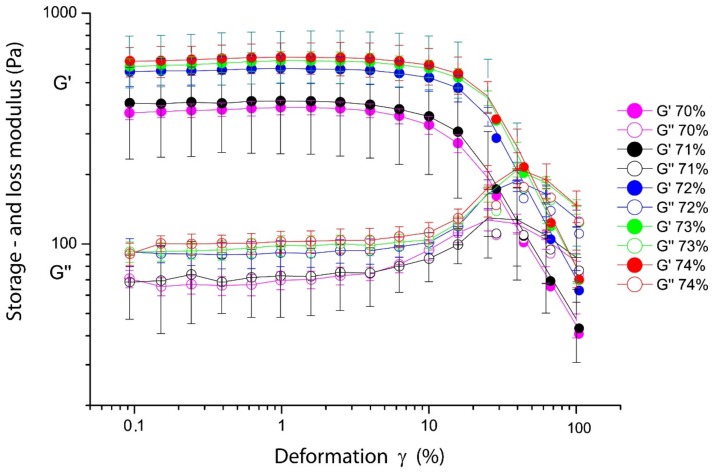
The storage (*G*’) and loss (*G’*’) modulus in amplitude sweeps of fresh milk emulsions from concentrations 70 to 74%.

Amplitude sweeps offer a tool to understand the structure, mechanical response and the dynamics under shear of the emulsion. Especially, the nonlinear regime offers deeper insight in the behaviour, and gives, in addition, some indications about the mouthfeel under oral processing of the emulsions. The results from the amplitude sweeps of emulsions prepared from fresh milk at various oil concentrations are shown in Figure 10.

The amplitude sweeps show three major features: First, they indicate a significant jump in the low-deformation storage modulus G’, a significant increase between the concentrations 70%, 71% to 72%, 73%, 74% by 200 Pa. The linear deformation regime ranges from 8 to 9% of deformation before, and second, a rapid shear thinning at amplitudes of 10% deformation appears, which corresponds to significant changes in the structure of the emulsion as will be discussed below. The same effect is indicated by the increase of the loss modulus G’’ around the deformation where G’ decreases.

**Figure 11:**
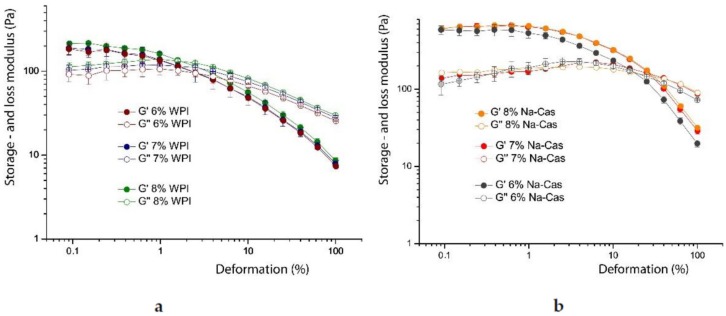
The storage (*G*’) and loss (*G’*’) modulus in amplitude sweeps of WPI (**a**) and Na-Cas (**b**) in the concentration range from 6 to 8%, each, with 73% oil in water.

The amplitude sweeps of the reconstructed emulsions show a different behaviour. In Figure 11, it is shown that between concentrations of 6 to 8% *w*/*w*, for both WPI and Na-Cas, the change of the moduli is marginal. The amplitude sweeps are practically identical within the error bars. In both cases the linear deformation regime (linear viscoelastic range) is significantly shorter, compared to emulsions of fresh milk, the decrease of the storage moduli is steeper, compared to fresh milk emulsions. Also, the behaviour of the loss modulus is quite different. It does not show a pronounced “hump” at deformations at the end of the linear viscoelastic regime. The storage modulus of emulsions stabilised with caseins is roughly an order of magnitude higher, than the storage modulus of purely WPI stabilised emulsions. This corresponds, again, with the change of the size of the oil droplets shown in Figure 4 and Figure 9.

#### 3.4.2. Frequency Sweeps

**Figure 12:**
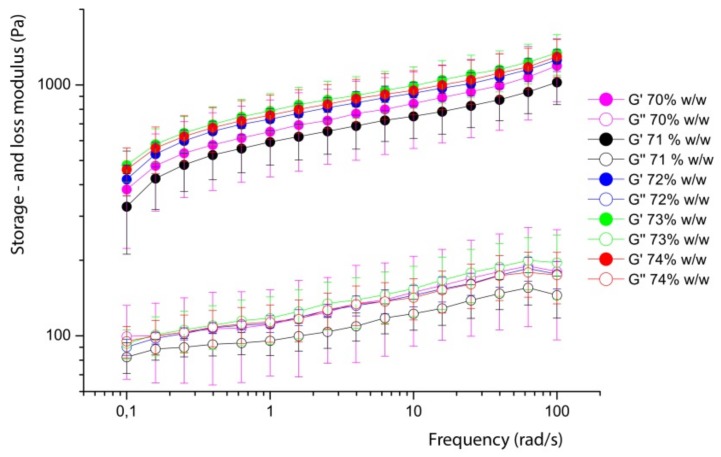
The storage (*G*’) and loss (*G’*’) modulus in frequency sweep of fresh milk emulsions from 70% *w*/*w* to 74% *w*/*w* oil at deformations γ = 0.5%.

The frequency sweeps indicate the time depended behaviour of the emulsions. First, the frequency sweep of the corresponding fresh milk emulsions are shown in Figure 12. 

**Figure 13:**
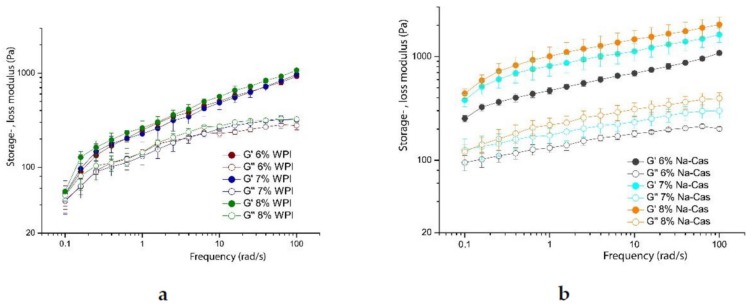
The storage (*G*’) and loss (*G’*’) modulus in freuqency sweeps of WPI (**a**) and Na-Cas (**b**) in the concentration range from 6 to 8%, each, with 73% oil in water at shear deformations γ = 0.5%.

Obviously, the different emulsions prepared by fresh milk behave very similar in the frequency range from 0.1 to 100 1/s. This is not surprising, as the shear deformation remains with 0.5% in the linear regime. More interesting is the comparison of this result with the reconstructed emulsions using WPI and Na-Cas as emulsifiers. These results are shown in Figure 13.

Figure 13a shows the graph of variously concentrated sodium caseinate emulsions. All emulsions have an oil content of 73% *w*/*w*. Emulsions containing 8% *w*/*w* sodium caseinate in the aqueous solution show the highest storage and loss modulus. In general, the elastic behaviour outweighs the viscous behaviour (G’ > G”) in all emulsions. A significantly lower storage and loss modulus shows emulsions stabilised by whey proteins (see Figure 13b). Both figures show the increase of the moduli with increasing frequency. The protein concentration also has a positive influence on the stability of the emulsion in the rheological properties. Thus, the emulsion with the highest protein concentration has the highest moduli, regardless of the use of milk protein-type fractions. Note the increase in the curves when comparing sodium caseinate emulsions and WPI emulsions. WPI emulsions show a greater slope than sodium caseinate emulsions. This indicates that WPI emulsions have a stronger effect on increasing frequency. It is also possible to draw conclusions about the temporal behaviour of the emulsions by means of the frequency sweeps. Thus, a slight change of the moduli in dependence of the frequency is an indication of the time stability. Sodium caseinate-stabilised emulsions, therefore, show higher time stability than WPI-stabilised emulsions. Comparing these results with those from the literature, similar courses are obtained with respect to the property of the higher moduli with increasing oil content [18]. The rheological properties between the different oils were also examined in a recent publication. Likewise, Mattia et al. [19] used a frequency of 0.11/s to 1001/s and obtained similar storage moduli to those measured in this experiment. However, egg proteins were used here, as in mayonnaise. The slight increase in G’ values was also observed here, as is also explained by the interactions between the droplets.

### 3.5. Thermal Properties of Milk Emulsions

**Figure 14:**
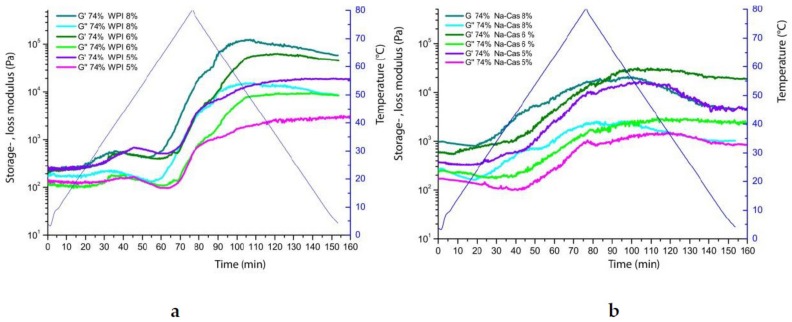
Storage modulus (*G*’) and loss (*G’*’) modulus and loss moduli of under temperature increase and decrease (4 °C to 80 °C to 4 °C) of reconstructed emulsions with 74% oil and increasing WPI (**a**), and Na-Cas (**b**) concentrations (from 5 to 8%).

Stable milk emulsions can be transformed into soft solids under heating. Consequently, they form a soft gel. To examine this liquid solid transition, rheological measurements under temperature ramps have been performed. The results are shown in Figure 14. 

The elastic part, G’, of the sample overlies the viscous portion, G’’, in all measurements, which is due to the stable emulsion and the jamming effect between the densely packed oil droplets. At the same time, a steady increase can be observed, intensified after reaching 80 °C, where G’ and G’’ increase further. It shows that there is an increasing firmer, gel-like structure caused by the heating of the sample. Upon heating, there are also interactions between adsorbed and non-adsorbed proteins and increasing strength of hydrophobic interactions [10].

The heating and subsequent cooling of the emulsions, by means a 4 °C to 80 °C to 4 °C temperature profile, show a strong change in the rheological properties of all investigated emulsions. The cause of this effect is the energy input due to temperature increase and thus the change in the structural properties of the proteins. Whey proteins are known for their sensitivity to heat, accompanied by changes in temperature (irreversible) alteration of the secondary and tertiary structures. Heating causes denaturation of the whey proteins, which in turn increases the adsorption and strength. The denaturation causes hydrophobic interactions and disulfide bonds, with which Chen and Dickinson explained the increasing G’ values of the whey protein emulsions as the temperature increases to 85 °C [20]. They also explain the increasing G’ values during cooling from 85 to 30 °C due to the enhanced hydrogen bonds. 

The apparent plateau value after heating the emulsions can be regarded as gelation, as the storage and loss modulus remains at a high level and does not drop again. Only in the sodium caseinate emulsions is a slight decrease of the modulus observed. This indicates that, due to the higher stability, the sodium caseinate is less responsive to heat-induced gelation than whey proteins. The storage modulus and loss modulus decrease slightly towards the end and the emulsion therefore softens and is not as stable as the gel structure formed by the WPI.

## 4. Discussion

**Figure 15:**
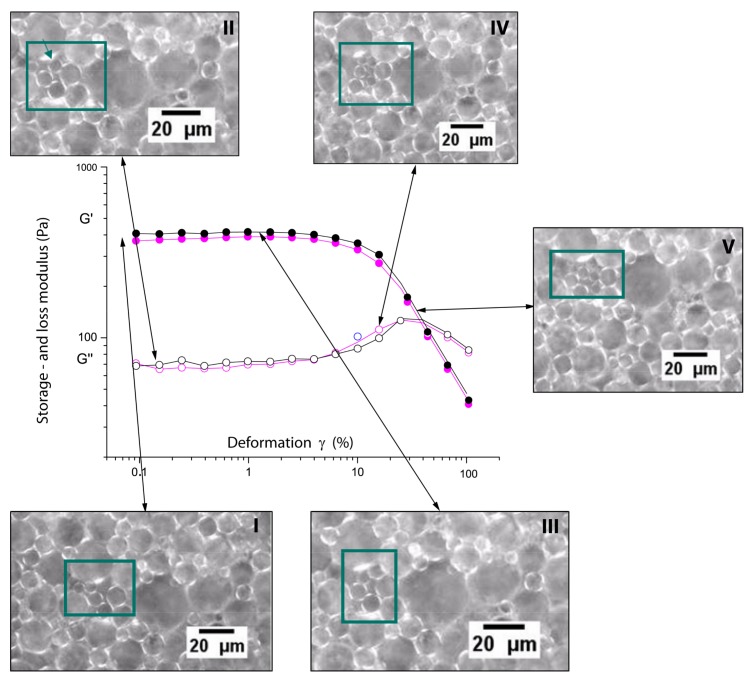
Rheomicroscopy observations for the storage (*G*’) and loss (*G’*’) modulus from fresh milk emulsions (70% oil) and snapshots at different deformations. The micrographs Ⅰ–Ⅴ correspond to different shear deformations and are indicated by the arrows. Visible changes are indicated by the rectangles in the photographs.

The key issue for understanding the physics of these dense milk emulsions is given by the rheological behaviour in connections with the microscopy. In the case of fresh milk, the “jump-like” increase of the storage modulus *G*’ between 70/71% and 72/73% (see Figure 10) compares directly to the corresponding microscopy shown in Figure 8, where the increasing concentration shows the nature of the jamming transition. At 65% oil concentration, the oil droplets appear even more spherical (and mobile), whereas at 73%, the oil droplets are more compact and show a more pronounced polyeder-like shape. The jamming cage is formed; the large droplets are immobile at shear deformations up to 10%, which corresponds to the well pronounced linear viscoelastic regime. Only at larger deformations does the jamming cage “break”; also, larger droplets can be locally rearranged. To confirm this, rheology and microscopy have been performed simultaneously by rheomicroscopic experiments. These results are summarised in Figure 15; it is clearly visible that during the linear viscoelastic regime, only small oil droplets (compare Figure 3) can “fill gaps” between larger droplets. Only when the shear deformation and its corresponding shear energy become larger than the cage energy and cage diameter (given by the interactions between the surfactants and the cage perimeter), the oil droplets rearrange and the storage modulus decreases. 

Picture Ⅰ in the figure shows the starting point, i.e., when no shear is applied. The figure resembles the corresponding microscopy results given in Figure 8. Picture Ⅱ show the early stages in the linear viscoelastic regime. Small oil droplets are already able to move. These motions represent local rearrangements only, consequently the modulus does not change and the response remains linear. Picture Ⅲ shows further motion of smaller droplet into gaps. The motions become more and more “directed”, and the droplets follow the direction of the shear. In picture Ⅳ, the gaps are filled more and more, and local deformations and rearrangements of the droplets become more unlikely, therefore the viscous part, expressed by the loss modulus G”, increases. In picture Ⅴ, more and more smaller droplets appear, cages are formed and reformed, rearrangements on larger scales begin and the storage modulus decreases.

The final point concerns the heat sensitivity of milk emulsions. Of course, pure milk does not gel under heating, but certainly whey proteins form under heating above 72 °C a network, well known as milk skin, which consists of denatured and cross-linked β-Lactoglobulins [21]. The crosslinking process is induced by forming disulphide bond of the amino acids nonbounded cysteine distributed along the signal peptide parts along the β-Lactoglobulins. The formation of the disulphide bridges is favoured at temperatures between 70 and 72 °C. The strong increase of the storage modulus during heating corresponds indeed to this temperature range. The number for reactive cysteine in casein chains is negligible, thus it is not surprising, that the contribution of casein to the modulus during heating is less pronounced.

However, the heat-sensitive whey proteins, mainly the β-Lactoglobulin, are very much restricted in the small water regions between the oil droplets. These water-soluble whey proteins are mainly squeezed and confined in the restricted space between the oil droplets, where their local concentration is much larger than their overlap concentration. The cross-link formation becomes very likely.

Open questions remain for the reconstructed emulsions prepared by whey protein isolates and caseins. The precise information about their molecular structure lacks completely. For fundamental research projects and the basic understanding of the physics on molecular scales, this information is essential to expanding understanding of the emulsification process, especially the adsorption on the liquid interface between oil and water droplets. The experiments investigating the droplet size suggest very different behaviour on the interfaces. Although the mainly (by the extraction and drying process) denatured WPI can still be considered a linear protein chain, the shape and structure of the different caseins remain unclear. However, it is likely that the strong hydrophobic α- and β-caseins are strongly collapsed in the solvent water and form tight globules, which adsorb rather densely at the surface of the oil droplets; whereas, the widely denatured whey proteins require more space on the surfaces in balancing entropy and energy at the interfaces. Then, the oil droplets remain larger than in the casein case. This clarification will require more research with better defined and fully characterised milk proteins.

## 5. Conclusions

The aim of this paper is to acquire insight in the emulsification properties of fresh, native, nonhomogenised milk vegetable oils, such as rapeseed oil produced with relatively “mild” shear rates, to maintain the native structure of the fat particles and the casein micelles (see, e.g., [4] for physical models). The phenomenological properties of the emulsions have been investigated with stability observations, particle size analysis, microscopy, rheology and rheo-optics. It was shown that indeed stable emulsion can be found in the range of 68 to 73% oil without adding further emulsifiers. The origin of the concentration range is given by by the limiting amount of natural emulsifiers in milk, i.e., the whey proteins and casein micelles. These differently shaped emulsifiers need to distribute themselves on the interface between the oil droplets and the water (whey) according to the Marangoni effect [22,23]. Additionally, the ratio between caseins and whey protein in natural milk will also limit the droplet size, due to their increasing surface/volume ratio.

Note that the smaller fat particles of fresh milk do not seem to play a significant role during the emulsification process; they integrate themselves in the emulsion. This is not surprising, as a biological membrane composed of lipids (and embedded membrane protein) surrounds them, which guarantees the preservation of their structure [4] as long as the shear forces during the emulsification processes are sufficiently small.

The rheo-optics experiments offer a direct observation of the jamming situation in these milk emulsions. The increasing shear deformation correlates directly with the “cage” and the particle size. Only at large shear deformations can shear thinning take place, as shown by the strong decrease of G’ (see, e.g., Figure 15), when highly cooperative motions are enabled.

Milk emulsions offer a broad range of new applications for emulsions based on natural food products, based on pure physical methods. Indeed, fresh (pasteurised) milk can be used as an option for sufficiently stable emulsions, as an alternative for egg yolk based emulsions. Especially in the culinary world milk emulsions offer novel ways for flavour and textures. The weak crosslinking under heating provides new ways of controlling the texture. The crosslinking is much weaker compared to emulsions used with egg white or egg yolk [5] and is especially interesting for emulsified soft gel-like milk creams prepared directly during pasteurisation.

## Figures and Tables

**Table 1:** Experimental parameters for the rheological experiments.

Parameters	Amplitude-Sweep	Frequency-Sweep	Temperature Profile
temperature (°C)	25	25	4–80–4
strain γ (%)	0.1–100	0.5	0.5
Angular frequency ω (rad/s)	10	0.1–100	10
